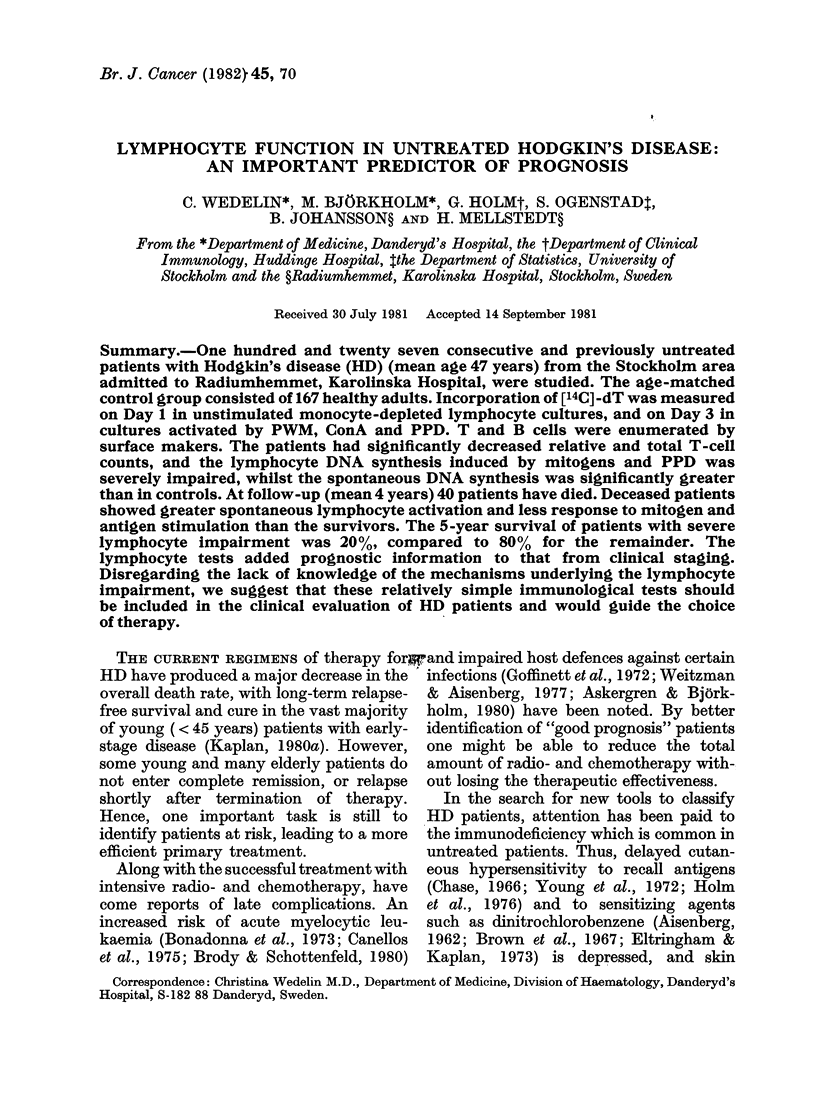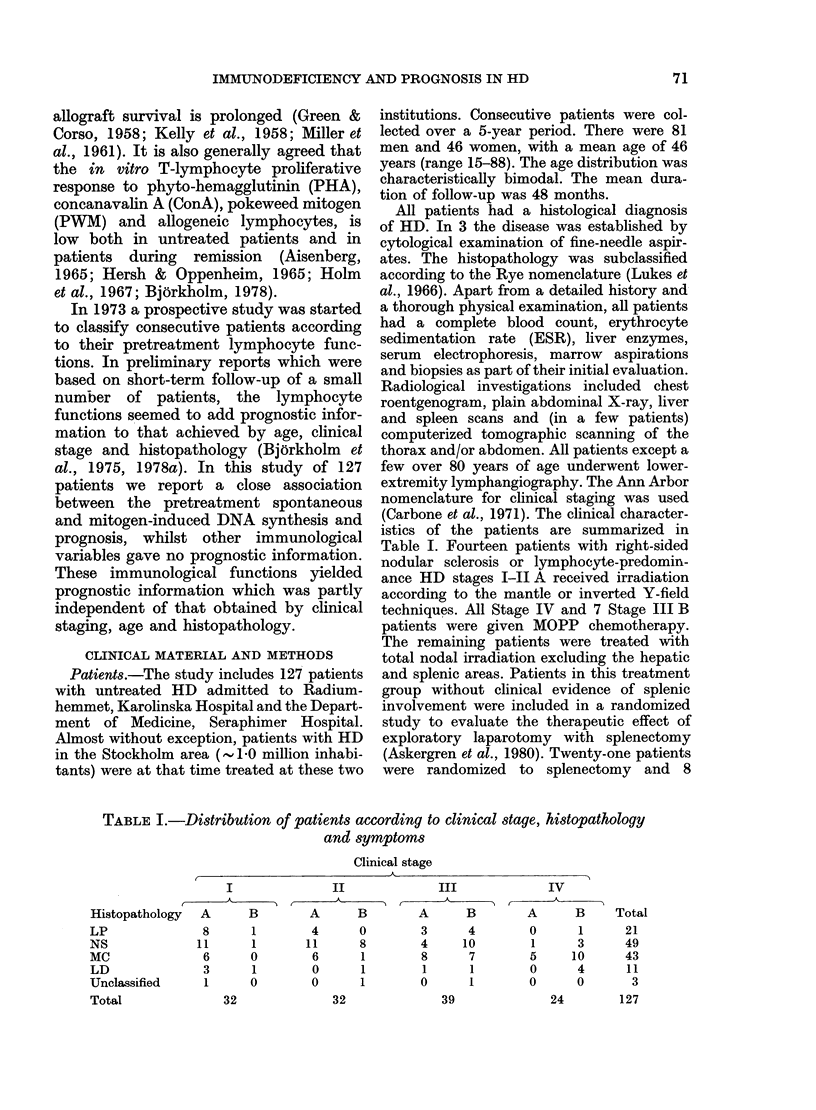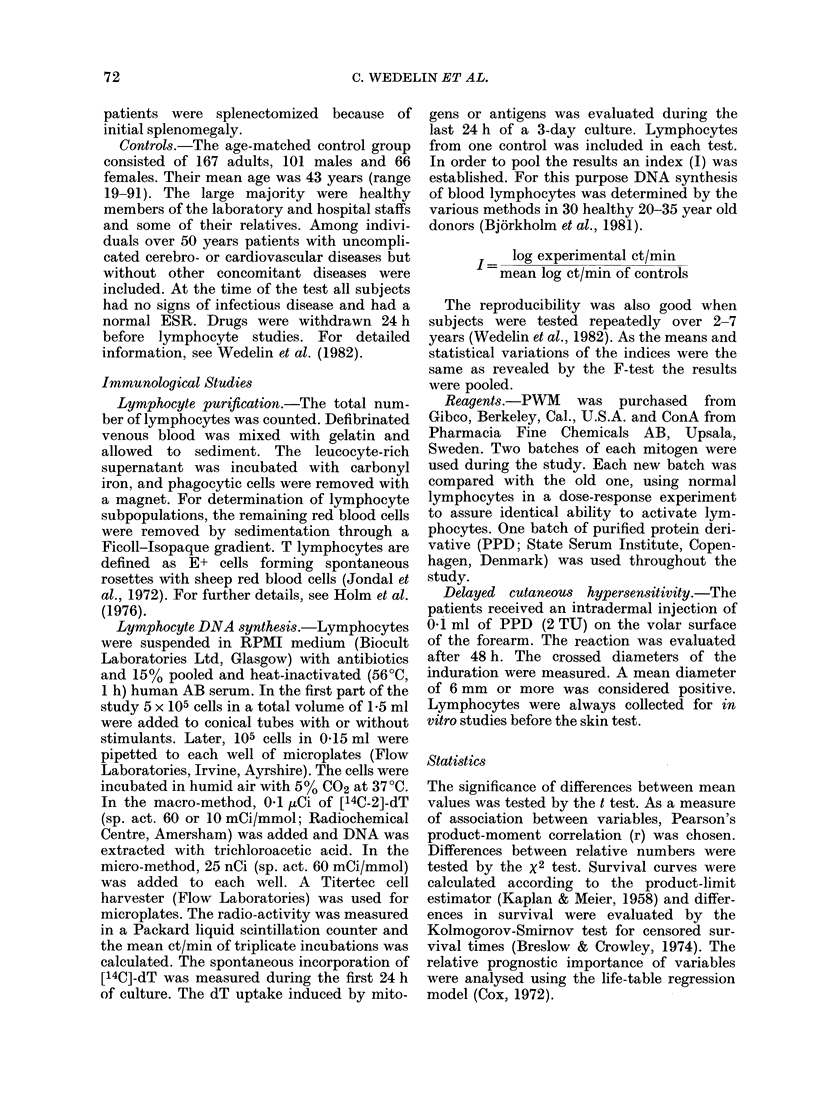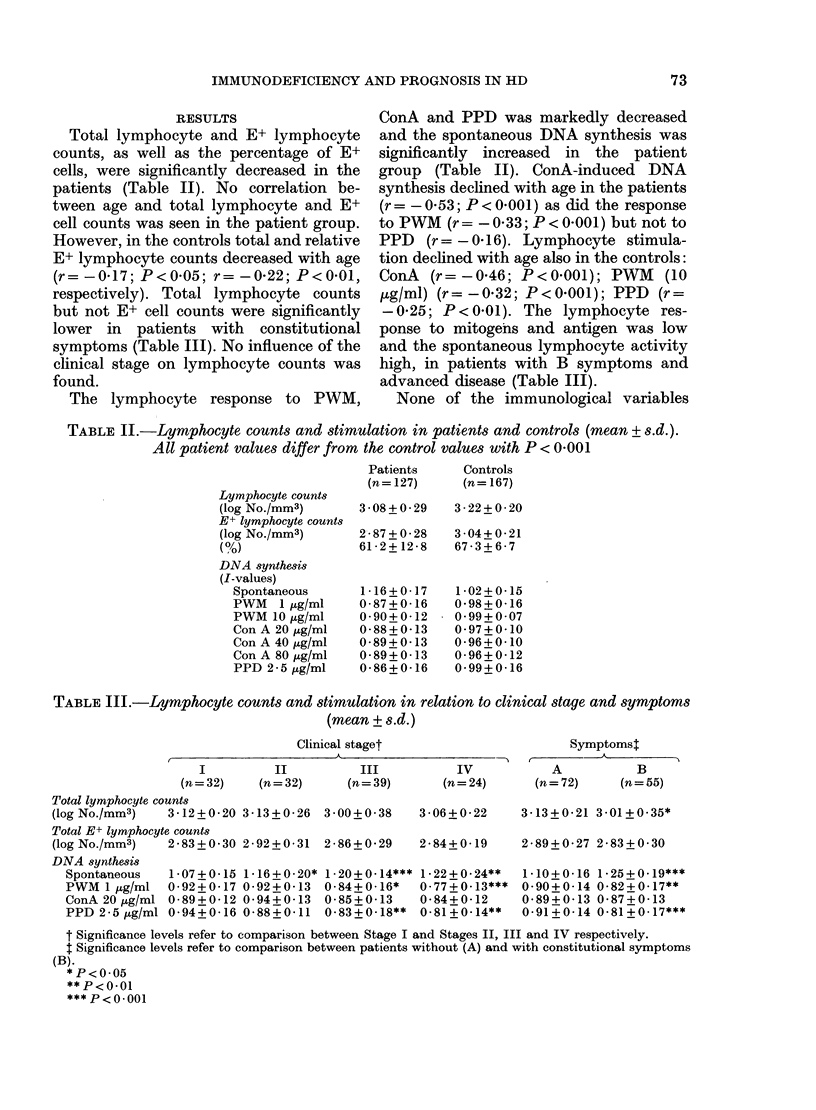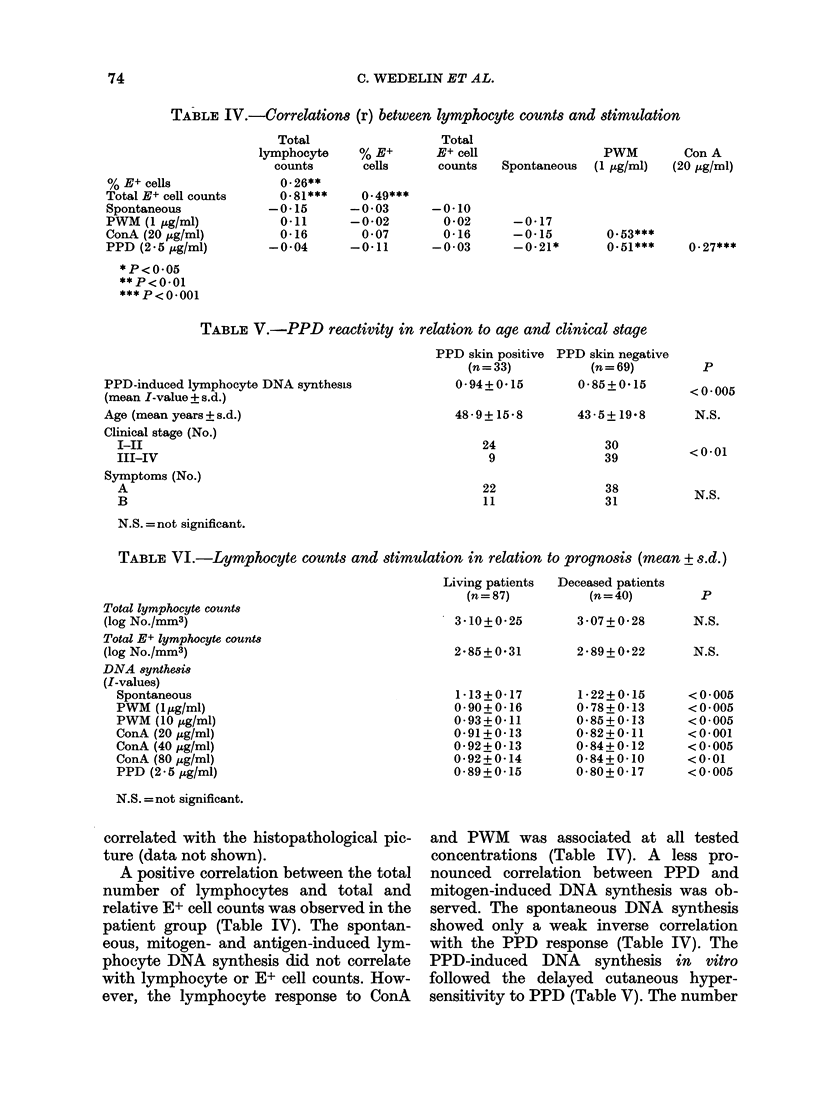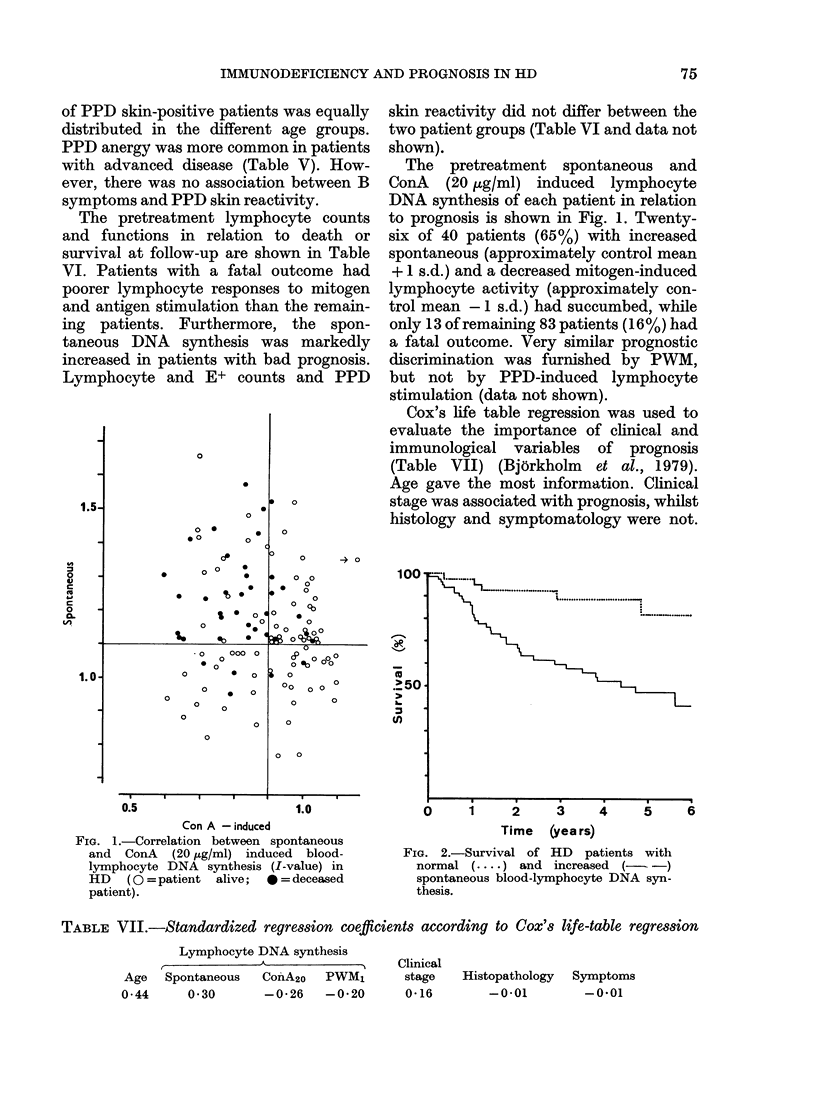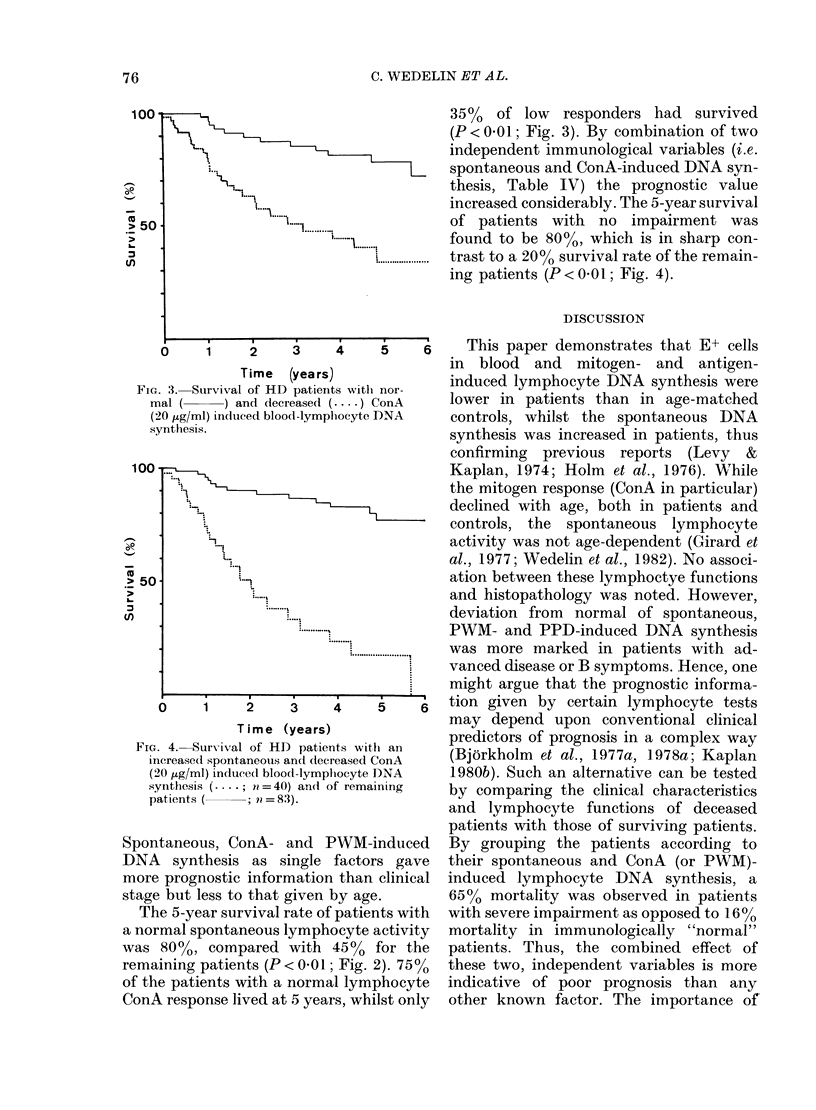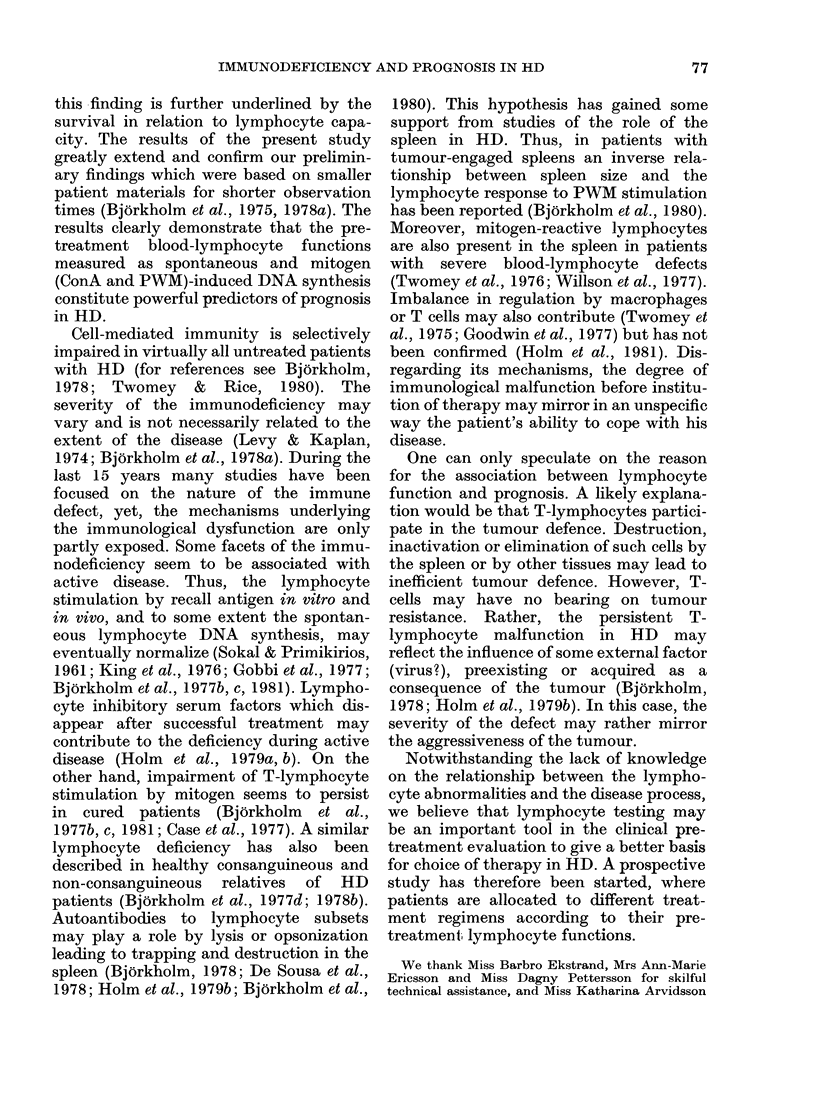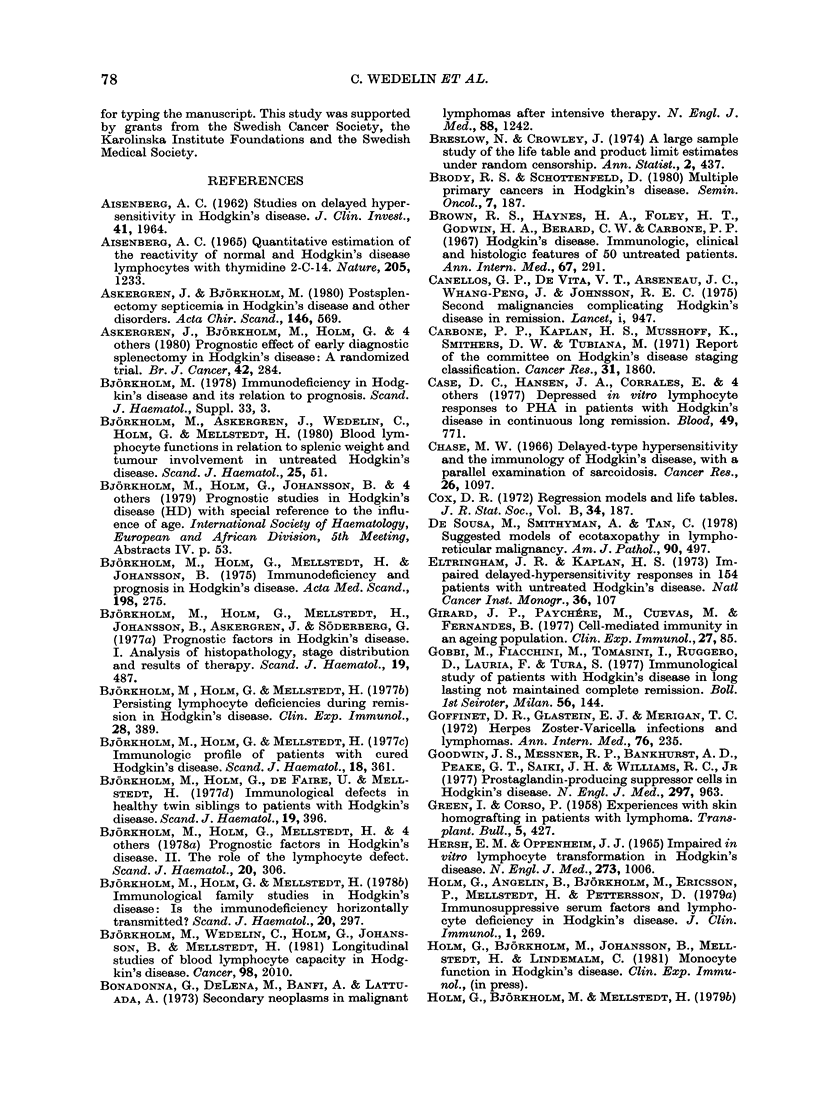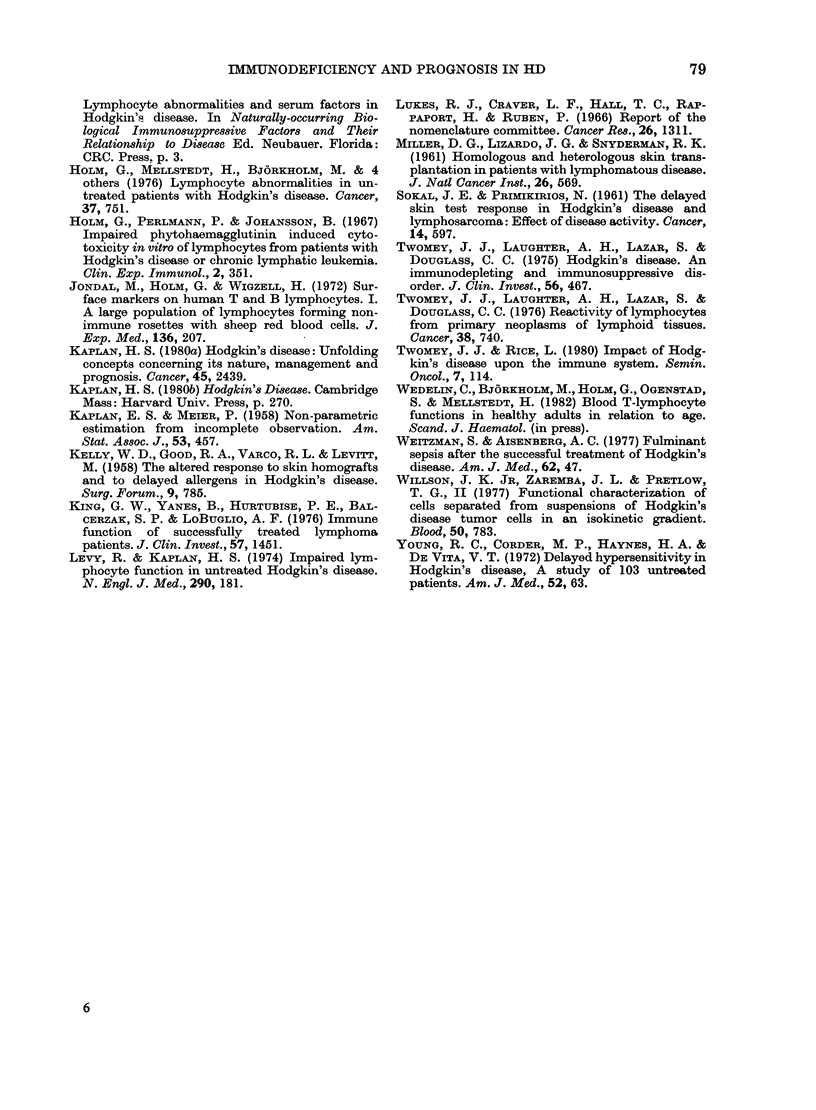# Lymphocyte function in untreated Hodgkin's disease: an important predictor of prognosis.

**DOI:** 10.1038/bjc.1982.9

**Published:** 1982-01

**Authors:** C. Wedelin, M. Björkholm, G. Holm, S. Ogenstad, B. Johansson, H. Mellstedt

## Abstract

One hundred and twenty seven consecutive and previously untreated patients with Hodgkin's disease (HD) (mean age 47 years) from the Stockholm area admitted to Radiumhemmet, Karolinska Hospital, were studied. The age-matched control group consisted of 167 healthy adults. Incorporation of [14C]-dT was measured on Day 1 in unstimulated monocyte-depleted lymphocyte cultures, and on Day 3 in cultures activated by PWM, ConA and PPD, T and B cells were enumerated by surface markers. The patients had significantly decreased relative and total T-cell counts, and the lymphocyte DNA synthesis induced by mitogens and PPD was severely impaired, whilst the spontaneous DNA synthesis was significantly greater than in controls. At follow-up (mean 4 years) 40 patients have died. Deceased patients showed greater spontaneous lymphocyte activation and less response to mitogen and antigen stimulation than the survivors. The 5-year survival of patients with severe lymphocyte impairment was 20%, compared to 80% for the remainder. The lymphocyte tests added prognostic information to that from clinical staging. Disregarding the lack of knowledge of the mechanisms underlying the lymphocyte impairment, we suggest that these relatively simple immunological tests should be included in the clinical evaluation of HD patients and would guide the choice of therapy.


Br. J. Cancer (1982) 45, 70

### LYMPHOCYTE FUNCTION IN UNTREATED HODGKIN'S DISEASE: AN IMPORTANT PREDICTOR OF PROGNOSIS

C. WEDELIN*, M. BJÖRKHOLM*, G. HOLM†, S. OGENSTAD‡, B. JOHANSSON§ AND H. MELLSTEDT§

*From the *Department of Medicine, Danderyd's Hospital, the †Department of Clinical Immunology, Huddinge Hospital, ‡the Department of Statistics, University of Stockholm and the §Radiumhemmet, Karolinska Hospital, Stockholm, Sweden*

Received 30 July 1981 Accepted 14 September 1981

**Summary.—One hundred and twenty seven consecutive and previously untreated patients with Hodgkin's disease (HD) (mean age 47 years) from the Stockholm area admitted to Radiumhemmet, Karolinska Hospital, were studied. The age-matched control group consisted of 167 healthy adults. Incorporation of [$^{14}$C]-dT was measured on Day 1 in unstimulated monocyte-depleted lymphocyte cultures, and on Day 3 in cultures activated by PWM, ConA and PPD. T and B cells were enumerated by surface makers. The patients had significantly decreased relative and total T-cell counts, and the lymphocyte DNA synthesis induced by mitogens and PPD was severely impaired, whilst the spontaneous DNA synthesis was significantly greater than in controls. At follow-up (mean 4 years) 40 patients have died. Deceased patients showed greater spontaneous lymphocyte activation and less response to mitogen and antigen stimulation than the survivors. The 5-year survival of patients with severe lymphocyte impairment was 20%, compared to 80% for the remainder. The lymphocyte tests added prognostic information to that from clinical staging. Disregarding the lack of knowledge of the mechanisms underlying the lymphocyte impairment, we suggest that these relatively simple immunological tests should be included in the clinical evaluation of HD patients and would guide the choice of therapy.**

THE CURRENT REGIMENS of therapy for HD have produced a major decrease in the overall death rate, with long-term relapse-free survival and cure in the vast majority of young (< 45 years) patients with early-stage disease (Kaplan, 1980*a*). However, some young and many elderly patients do not enter complete remission, or relapse shortly after termination of therapy. Hence, one important task is still to identify patients at risk, leading to a more efficient primary treatment.

Along with the successful treatment with intensive radio- and chemotherapy, have come reports of late complications. An increased risk of acute myelocytic leukaemia (Bonadonna *et al.*, 1973; Canellos *et al.*, 1975; Brody & Schottenfeld, 1980) and impaired host defences against certain infections (Goffinett *et al.*, 1972; Weitzman & Aisenberg, 1977; Askergren & Björkholm, 1980) have been noted. By better identification of "good prognosis" patients one might be able to reduce the total amount of radio- and chemotherapy without losing the therapeutic effectiveness.

In the search for new tools to classify HD patients, attention has been paid to the immunodeficiency which is common in untreated patients. Thus, delayed cutaneous hypersensitivity to recall antigens (Chase, 1966; Young *et al.*, 1972; Holm *et al.*, 1976) and to sensitizing agents such as dinitrochlorobenzene (Aisenberg, 1962; Brown *et al.*, 1967; Eltringham & Kaplan, 1973) is depressed, and skin

Correspondence: Christina Wedelin M.D., Department of Medicine, Division of Haematology, Danderyd's Hospital, S-182 88 Danderyd, Sweden.

allograft survival is prolonged (Green & Corso, 1958; Kelly *et al.*, 1958; Miller *et al.*, 1961). It is also generally agreed that the *in vitro* T-lymphocyte proliferative response to phyto-hemagglutinin (PHA), concanavalin A (ConA), pokeweed mitogen (PWM) and allogeneic lymphocytes, is low both in untreated patients and in patients during remission (Aisenberg, 1965; Hersh & Oppenheim, 1965; Holm *et al.*, 1967; Björkholm, 1978).

In 1973 a prospective study was started to classify consecutive patients according to their pretreatment lymphocyte functions. In preliminary reports which were based on short-term follow-up of a small number of patients, the lymphocyte functions seemed to add prognostic information to that achieved by age, clinical stage and histopathology (Björkholm *et al.*, 1975, 1978*a*). In this study of 127 patients we report a close association between the pretreatment spontaneous and mitogen-induced DNA synthesis and prognosis, whilst other immunological variables gave no prognostic information. These immunological functions yielded prognostic information which was partly independent of that obtained by clinical staging, age and histopathology.

### CLINICAL MATERIAL AND METHODS

*Patients.*—The study includes 127 patients with untreated HD admitted to Radiumhemmet, Karolinska Hospital and the Department of Medicine, Seraphimer Hospital. Almost without exception, patients with HD in the Stockholm area ($\sim$1·0 million inhabitants) were at that time treated at these two institutions. Consecutive patients were collected over a 5-year period. There were 81 men and 46 women, with a mean age of 46 years (range 15–88). The age distribution was characteristically bimodal. The mean duration of follow-up was 48 months.

All patients had a histological diagnosis of HD. In 3 the disease was established by cytological examination of fine-needle aspirates. The histopathology was subclassified according to the Rye nomenclature (Lukes *et al.*, 1966). Apart from a detailed history and a thorough physical examination, all patients had a complete blood count, erythrocyte sedimentation rate (ESR), liver enzymes, serum electrophoresis, marrow aspirations and biopsies as part of their initial evaluation. Radiological investigations included chest roentgenogram, plain abdominal X-ray, liver and spleen scans and (in a few patients) computerized tomographic scanning of the thorax and/or abdomen. All patients except a few over 80 years of age underwent lower-extremity lymphangiography. The Ann Arbor nomenclature for clinical staging was used (Carbone *et al.*, 1971). The clinical characteristics of the patients are summarized in Table I. Fourteen patients with right-sided nodular sclerosis or lymphocyte-predominance HD stages I–II A received irradiation according to the mantle or inverted Y-field techniques. All Stage IV and 7 Stage III B patients were given MOPP chemotherapy. The remaining patients were treated with total nodal irradiation excluding the hepatic and splenic areas. Patients in this treatment group without clinical evidence of splenic involvement were included in a randomized study to evaluate the therapeutic effect of exploratory laparotomy with splenectomy (Askergren *et al.*, 1980). Twenty-one patients were randomized to splenectomy and 8

TABLE I.—*Distribution of patients according to clinical stage, histopathology and symptoms*

| | Clinical stage | | | | | | | | |
|---|---|---|---|---|---|---|---|---|---|
| | I | | II | | III | | IV | | |
| Histopathology | A | B | A | B | A | B | A | B | Total |
| LP | 8 | 1 | 4 | 0 | 3 | 4 | 0 | 1 | 21 |
| NS | 11 | 1 | 11 | 8 | 4 | 10 | 1 | 3 | 49 |
| MC | 6 | 0 | 6 | 1 | 8 | 7 | 5 | 10 | 43 |
| LD | 3 | 1 | 0 | 1 | 1 | 1 | 0 | 4 | 11 |
| Unclassified | 1 | 0 | 0 | 1 | 0 | 1 | 0 | 0 | 3 |
| Total | 32 | | 32 | | 39 | | 24 | | 127 |

patients were splenectomized because of initial splenomegaly.

*Controls.*—The age-matched control group consisted of 167 adults, 101 males and 66 females. Their mean age was 43 years (range 19–91). The large majority were healthy members of the laboratory and hospital staffs and some of their relatives. Among individuals over 50 years patients with uncomplicated cerebro- or cardiovascular diseases but without other concomitant diseases were included. At the time of the test all subjects had no signs of infectious disease and had a normal ESR. Drugs were withdrawn 24 h before lymphocyte studies. For detailed information, see Wedelin *et al.* (1982).

### *Immunological Studies*

*Lymphocyte purification.*—The total number of lymphocytes was counted. Defibrinated venous blood was mixed with gelatin and allowed to sediment. The leucocyte-rich supernatant was incubated with carbonyl iron, and phagocytic cells were removed with a magnet. For determination of lymphocyte subpopulations, the remaining red blood cells were removed by sedimentation through a Ficoll–Isopaque gradient. T lymphocytes are defined as $E^{+}$ cells forming spontaneous rosettes with sheep red blood cells (Jondal *et al.*, 1972). For further details, see Holm *et al.* (1976).

*Lymphocyte DNA synthesis.*—Lymphocytes were suspended in RPMI medium (Biocult Laboratories Ltd, Glasgow) with antibiotics and 15% pooled and heat-inactivated (56 °C, 1 h) human AB serum. In the first part of the study $5 \times 10^5$ cells in a total volume of 1·5 ml were added to conical tubes with or without stimulants. Later, $10^5$ cells in 0·15 ml were pipetted to each well of microplates (Flow Laboratories, Irvine, Ayrshire). The cells were incubated in humid air with 5% $CO_2$ at 37 °C. In the macro-method, 0·1 $\mu$Ci of [$^{14}$C-2]-dT (sp. act. 60 or 10 mCi/mmol; Radiochemical Centre, Amersham) was added and DNA was extracted with trichloroacetic acid. In the micro-method, 25 nCi (sp. act. 60 mCi/mmol) was added to each well. A Titertec cell harvester (Flow Laboratories) was used for microplates. The radio-activity was measured in a Packard liquid scintillation counter and the mean ct/min of triplicate incubations was calculated. The spontaneous incorporation of [$^{14}$C]-dT was measured during the first 24 h of culture. The dT uptake induced by mitogens or antigens was evaluated during the last 24 h of a 3-day culture. Lymphocytes from one control was included in each test. In order to pool the results an index (I) was established. For this purpose DNA synthesis of blood lymphocytes was determined by the various methods in 30 healthy 20–35 year old donors (Björkholm *et al.*, 1981).

$$I = \frac{\text{log experimental ct/min}}{\text{mean log ct/min of controls}}$$

The reproducibility was also good when subjects were tested repeatedly over 2–7 years (Wedelin *et al.*, 1982). As the means and statistical variations of the indices were the same as revealed by the F-test the results were pooled.

*Reagents.*—PWM was purchased from Gibco, Berkeley, Cal., U.S.A. and ConA from Pharmacia Fine Chemicals AB, Upsala, Sweden. Two batches of each mitogen were used during the study. Each new batch was compared with the old one, using normal lymphocytes in a dose-response experiment to assure identical ability to activate lymphocytes. One batch of purified protein derivative (PPD; State Serum Institute, Copenhagen, Denmark) was used throughout the study.

*Delayed cutaneous hypersensitivity.*—The patients received an intradermal injection of 0·1 ml of PPD (2 TU) on the volar surface of the forearm. The reaction was evaluated after 48 h. The crossed diameters of the induration were measured. A mean diameter of 6 mm or more was considered positive. Lymphocytes were always collected for *in vitro* studies before the skin test.

### *Statistics*

The significance of differences between mean values was tested by the *t* test. As a measure of association between variables, Pearson's product-moment correlation (r) was chosen. Differences between relative numbers were tested by the $\chi^2$ test. Survival curves were calculated according to the product-limit estimator (Kaplan & Meier, 1958) and differences in survival were evaluated by the Kolmogorov-Smirnov test for censored survival times (Breslow & Crowley, 1974). The relative prognostic importance of variables were analysed using the life-table regression model (Cox, 1972).

### RESULTS

Total lymphocyte and $E^+$ lymphocyte counts, as well as the percentage of $E^+$ cells, were significantly decreased in the patients (Table II). No correlation between age and total lymphocyte and $E^+$ cell counts was seen in the patient group. However, in the controls total and relative $E^+$ lymphocyte counts decreased with age ($r = -0{\cdot}17$; $P < 0{\cdot}05$; $r = -0{\cdot}22$; $P < 0{\cdot}01$, respectively). Total lymphocyte counts but not $E^+$ cell counts were significantly lower in patients with constitutional symptoms (Table III). No influence of the clinical stage on lymphocyte counts was found.

The lymphocyte response to PWM, ConA and PPD was markedly decreased and the spontaneous DNA synthesis was significantly increased in the patient group (Table II). ConA-induced DNA synthesis declined with age in the patients ($r = -0{\cdot}53$; $P < 0{\cdot}001$) as did the response to PWM ($r = -0{\cdot}33$; $P < 0{\cdot}001$) but not to PPD ($r = -0{\cdot}16$). Lymphocyte stimulation declined with age also in the controls: ConA ($r = -0{\cdot}46$; $P < 0{\cdot}001$); PWM (10 μg/ml) ($r = -0{\cdot}32$; $P < 0{\cdot}001$); PPD ($r = -0{\cdot}25$; $P < 0{\cdot}01$). The lymphocyte response to mitogens and antigen was low and the spontaneous lymphocyte activity high, in patients with B symptoms and advanced disease (Table III).

None of the immunological variables

TABLE II.—*Lymphocyte counts and stimulation in patients and controls (mean ± s.d.). All patient values differ from the control values with* $P < 0{\cdot}001$

| | Patients ($n = 127$) | Controls ($n = 167$) |
|---|---|---|
| *Lymphocyte counts* | | |
| (log No./mm³) | 3·08±0·29 | 3·22±0·20 |
| *$E^+$ lymphocyte counts* | | |
| (log No./mm³) | 2·87±0·28 | 3·04±0·21 |
| (%) | 61·2±12·8 | 67·3±6·7 |
| *DNA synthesis* | | |
| (*I*-values) | | |
| Spontaneous | 1·16±0·17 | 1·02±0·15 |
| PWM 1 μg/ml | 0·87±0·16 | 0·98±0·16 |
| PWM 10 μg/ml | 0·90±0·12 | 0·99±0·07 |
| Con A 20 μg/ml | 0·88±0·13 | 0·97±0·10 |
| Con A 40 μg/ml | 0·89±0·13 | 0·96±0·10 |
| Con A 80 μg/ml | 0·89±0·13 | 0·96±0·12 |
| PPD 2·5 μg/ml | 0·86±0·16 | 0·99±0·16 |

TABLE III.—*Lymphocyte counts and stimulation in relation to clinical stage and symptoms (mean ± s.d.)*

| | Clinical stage† | | | | Symptoms‡ | |
|---|---|---|---|---|---|---|
| | I ($n=32$) | II ($n=32$) | III ($n=39$) | IV ($n=24$) | A ($n=72$) | B ($n=55$) |
| *Total lymphocyte counts* | | | | | | |
| (log No./mm³) | 3·12±0·20 | 3·13±0·26 | 3·00±0·38 | 3·06±0·22 | 3·13±0·21 | 3·01±0·35* |
| *Total $E^+$ lymphocyte counts* | | | | | | |
| (log No./mm³) | 2·83±0·30 | 2·92±0·31 | 2·86±0·29 | 2·84±0·19 | 2·89±0·27 | 2·83±0·30 |
| *DNA synthesis* | | | | | | |
| Spontaneous | 1·07±0·15 | 1·16±0·20* | 1·20±0·14*** | 1·22±0·24** | 1·10±0·16 | 1·25±0·19*** |
| PWM 1 μg/ml | 0·92±0·17 | 0·92±0·13 | 0·84±0·16* | 0·77±0·13*** | 0·90±0·14 | 0·82±0·17** |
| ConA 20 μg/ml | 0·89±0·12 | 0·94±0·13 | 0·85±0·13 | 0·84±0·12 | 0·89±0·13 | 0·87±0·13 |
| PPD 2·5 μg/ml | 0·94±0·16 | 0·88±0·11 | 0·83±0·18** | 0·81±0·14** | 0·91±0·14 | 0·81±0·17*** |

† Significance levels refer to comparison between Stage I and Stages II, III and IV respectively.
‡ Significance levels refer to comparison between patients without (A) and with constitutional symptoms (B).
* $P < 0{\cdot}05$
** $P < 0{\cdot}01$
*** $P < 0{\cdot}001$

TABLE IV.—*Correlations* (r) *between lymphocyte counts and stimulation*

| | Total lymphocyte counts | % $E^+$ cells | Total $E^+$ cell counts | Spontaneous | PWM (1 μg/ml) | Con A (20 μg/ml) |
|---|---|---|---|---|---|---|
| % $E^+$ cells | 0·26** | | | | | |
| Total $E^+$ cell counts | 0·81*** | 0·49*** | | | | |
| Spontaneous | −0·15 | −0·03 | −0·10 | | | |
| PWM (1 μg/ml) | 0·11 | −0·02 | 0·02 | −0·17 | | |
| ConA (20 μg/ml) | 0·16 | 0·07 | 0·16 | −0·15 | 0·53*** | |
| PPD (2·5 μg/ml) | −0·04 | −0·11 | −0·03 | −0·21* | 0·51*** | 0·27*** |

* $P < 0·05$
** $P < 0·01$
*** $P < 0·001$

TABLE V.—*PPD reactivity in relation to age and clinical stage*

| | PPD skin positive ($n = 33$) | PPD skin negative ($n = 69$) | $P$ |
|---|---|---|---|
| PPD-induced lymphocyte DNA synthesis (mean $I$-value ± s.d.) | 0·94 ± 0·15 | 0·85 ± 0·15 | < 0·005 |
| Age (mean years ± s.d.) | 48·9 ± 15·8 | 43·5 ± 19·8 | N.S. |
| Clinical stage (No.) | | | |
| I–II | 24 | 30 | < 0·01 |
| III–IV | 9 | 39 | |
| Symptoms (No.) | | | |
| A | 22 | 38 | N.S. |
| B | 11 | 31 | |

N.S. = not significant.

TABLE VI.—*Lymphocyte counts and stimulation in relation to prognosis (mean ± s.d.)*

| | Living patients ($n = 87$) | Deceased patients ($n = 40$) | $P$ |
|---|---|---|---|
| *Total lymphocyte counts* (log No./mm³) | 3·10 ± 0·25 | 3·07 ± 0·28 | N.S. |
| *Total $E^+$ lymphocyte counts* (log No./mm³) | 2·85 ± 0·31 | 2·89 ± 0·22 | N.S. |
| *DNA synthesis* ($I$-values) | | | |
| Spontaneous | 1·13 ± 0·17 | 1·22 ± 0·15 | < 0·005 |
| PWM (1 μg/ml) | 0·90 ± 0·16 | 0·78 ± 0·13 | < 0·005 |
| PWM (10 μg/ml) | 0·93 ± 0·11 | 0·85 ± 0·13 | < 0·005 |
| ConA (20 μg/ml) | 0·91 ± 0·13 | 0·82 ± 0·11 | < 0·001 |
| ConA (40 μg/ml) | 0·92 ± 0·13 | 0·84 ± 0·12 | < 0·005 |
| ConA (80 μg/ml) | 0·92 ± 0·14 | 0·84 ± 0·10 | < 0·01 |
| PPD (2·5 μg/ml) | 0·89 ± 0·15 | 0·80 ± 0·17 | < 0·005 |

N.S. = not significant.

correlated with the histopathological picture (data not shown).

A positive correlation between the total number of lymphocytes and total and relative $E^+$ cell counts was observed in the patient group (Table IV). The spontaneous, mitogen- and antigen-induced lymphocyte DNA synthesis did not correlate with lymphocyte or $E^+$ cell counts. However, the lymphocyte response to ConA and PWM was associated at all tested concentrations (Table IV). A less pronounced correlation between PPD and mitogen-induced DNA synthesis was observed. The spontaneous DNA synthesis showed only a weak inverse correlation with the PPD response (Table IV). The PPD-induced DNA synthesis *in vitro* followed the delayed cutaneous hypersensitivity to PPD (Table V). The number of PPD skin-positive patients was equally distributed in the different age groups. PPD anergy was more common in patients with advanced disease (Table V). However, there was no association between B symptoms and PPD skin reactivity.

The pretreatment lymphocyte counts and functions in relation to death or survival at follow-up are shown in Table VI. Patients with a fatal outcome had poorer lymphocyte responses to mitogen and antigen stimulation than the remaining patients. Furthermore, the spontaneous DNA synthesis was markedly increased in patients with bad prognosis. Lymphocyte and $E^+$ counts and PPD skin reactivity did not differ between the two patient groups (Table VI and data not shown).

The pretreatment spontaneous and ConA (20 μg/ml) induced lymphocyte DNA synthesis of each patient in relation to prognosis is shown in Fig. 1. Twenty-six of 40 patients (65%) with increased spontaneous (approximately control mean + 1 s.d.) and a decreased mitogen-induced lymphocyte activity (approximately control mean − 1 s.d.) had succumbed, while only 13 of remaining 83 patients (16%) had a fatal outcome. Very similar prognostic discrimination was furnished by PWM, but not by PPD-induced lymphocyte stimulation (data not shown).

Cox's life table regression was used to evaluate the importance of clinical and immunological variables of prognosis (Table VII) (Björkholm *et al.*, 1979). Age gave the most information. Clinical stage was associated with prognosis, whilst histology and symptomatology were not.

Fig. 1.—Correlation between spontaneous and ConA (20 μg/ml) induced blood-lymphocyte DNA synthesis (*I*-value) in HD (○ = patient alive; ● = deceased patient).

Fig. 2.—Survival of HD patients with normal (....) and increased (——) spontaneous blood-lymphocyte DNA synthesis.

Table VII.—*Standardized regression coefficients according to Cox's life-table regression*

| Age | Lymphocyte DNA synthesis: Spontaneous | ConA20 | PWM1 | Clinical stage | Histopathology | Symptoms |
|---|---|---|---|---|---|---|
| 0·44 | 0·30 | −0·26 | −0·20 | 0·16 | −0·01 | −0·01 |

FIG. 3.—Survival of HD patients with normal (———) and decreased (. . . .) ConA (20 μg/ml) induced blood-lymphocyte DNA synthesis.

FIG. 4.—Survival of HD patients with an increased spontaneous and decreased ConA (20 μg/ml) induced blood-lymphocyte DNA synthesis (. . . . ; $n = 40$) and of remaining patients (——— ; $n = 83$).

Spontaneous, ConA- and PWM-induced DNA synthesis as single factors gave more prognostic information than clinical stage but less to that given by age.

The 5-year survival rate of patients with a normal spontaneous lymphocyte activity was 80%, compared with 45% for the remaining patients ($P < 0{\cdot}01$; Fig. 2). 75% of the patients with a normal lymphocyte ConA response lived at 5 years, whilst only 35% of low responders had survived ($P < 0{\cdot}01$; Fig. 3). By combination of two independent immunological variables (*i.e.* spontaneous and ConA-induced DNA synthesis, Table IV) the prognostic value increased considerably. The 5-year survival of patients with no impairment was found to be 80%, which is in sharp contrast to a 20% survival rate of the remaining patients ($P < 0{\cdot}01$; Fig. 4).

### DISCUSSION

This paper demonstrates that $E^+$ cells in blood and mitogen- and antigen-induced lymphocyte DNA synthesis were lower in patients than in age-matched controls, whilst the spontaneous DNA synthesis was increased in patients, thus confirming previous reports (Levy & Kaplan, 1974; Holm *et al.*, 1976). While the mitogen response (ConA in particular) declined with age, both in patients and controls, the spontaneous lymphocyte activity was not age-dependent (Girard *et al.*, 1977; Wedelin *et al.*, 1982). No association between these lymphoctye functions and histopathology was noted. However, deviation from normal of spontaneous, PWM- and PPD-induced DNA synthesis was more marked in patients with advanced disease or B symptoms. Hence, one might argue that the prognostic information given by certain lymphocyte tests may depend upon conventional clinical predictors of prognosis in a complex way (Björkholm *et al.*, 1977*a*, 1978*a*; Kaplan 1980*b*). Such an alternative can be tested by comparing the clinical characteristics and lymphocyte functions of deceased patients with those of surviving patients. By grouping the patients according to their spontaneous and ConA (or PWM)-induced lymphocyte DNA synthesis, a 65% mortality was observed in patients with severe impairment as opposed to 16% mortality in immunologically "normal" patients. Thus, the combined effect of these two, independent variables is more indicative of poor prognosis than any other known factor. The importance of this finding is further underlined by the survival in relation to lymphocyte capacity. The results of the present study greatly extend and confirm our preliminary findings which were based on smaller patient materials for shorter observation times (Björkholm *et al.*, 1975, 1978*a*). The results clearly demonstrate that the pretreatment blood-lymphocyte functions measured as spontaneous and mitogen (ConA and PWM)-induced DNA synthesis constitute powerful predictors of prognosis in HD.

Cell-mediated immunity is selectively impaired in virtually all untreated patients with HD (for references see Björkholm, 1978; Twomey & Rice, 1980). The severity of the immunodeficiency may vary and is not necessarily related to the extent of the disease (Levy & Kaplan, 1974; Björkholm *et al.*, 1978*a*). During the last 15 years many studies have been focused on the nature of the immune defect, yet, the mechanisms underlying the immunological dysfunction are only partly exposed. Some facets of the immunodeficiency seem to be associated with active disease. Thus, the lymphocyte stimulation by recall antigen *in vitro* and *in vivo*, and to some extent the spontaneous lymphocyte DNA synthesis, may eventually normalize (Sokal & Primikirios, 1961; King *et al.*, 1976; Gobbi *et al.*, 1977; Björkholm *et al.*, 1977*b*, *c*, 1981). Lymphocyte inhibitory serum factors which disappear after successful treatment may contribute to the deficiency during active disease (Holm *et al.*, 1979*a*, *b*). On the other hand, impairment of T-lymphocyte stimulation by mitogen seems to persist in cured patients (Björkholm *et al.*, 1977*b*, *c*, 1981; Case *et al.*, 1977). A similar lymphocyte deficiency has also been described in healthy consanguineous and non-consanguineous relatives of HD patients (Björkholm *et al.*, 1977*d*; 1978*b*). Autoantibodies to lymphocyte subsets may play a role by lysis or opsonization leading to trapping and destruction in the spleen (Björkholm, 1978; De Sousa *et al.*, 1978; Holm *et al.*, 1979*b*; Björkholm *et al.*, 1980). This hypothesis has gained some support from studies of the role of the spleen in HD. Thus, in patients with tumour-engaged spleens an inverse relationship between spleen size and the lymphocyte response to PWM stimulation has been reported (Björkholm *et al.*, 1980). Moreover, mitogen-reactive lymphocytes are also present in the spleen in patients with severe blood-lymphocyte defects (Twomey *et al.*, 1976; Willson *et al.*, 1977). Imbalance in regulation by macrophages or T cells may also contribute (Twomey *et al.*, 1975; Goodwin *et al.*, 1977) but has not been confirmed (Holm *et al.*, 1981). Disregarding its mechanisms, the degree of immunological malfunction before institution of therapy may mirror in an unspecific way the patient's ability to cope with his disease.

One can only speculate on the reason for the association between lymphocyte function and prognosis. A likely explanation would be that T-lymphocytes participate in the tumour defence. Destruction, inactivation or elimination of such cells by the spleen or by other tissues may lead to inefficient tumour defence. However, T-cells may have no bearing on tumour resistance. Rather, the persistent T-lymphocyte malfunction in HD may reflect the influence of some external factor (virus?), preexisting or acquired as a consequence of the tumour (Björkholm, 1978; Holm *et al.*, 1979*b*). In this case, the severity of the defect may rather mirror the aggressiveness of the tumour.

Notwithstanding the lack of knowledge on the relationship between the lymphocyte abnormalities and the disease process, we believe that lymphocyte testing may be an important tool in the clinical pretreatment evaluation to give a better basis for choice of therapy in HD. A prospective study has therefore been started, where patients are allocated to different treatment regimens according to their pretreatment lymphocyte functions.

We thank Miss Barbro Ekstrand, Mrs Ann-Marie Ericsson and Miss Dagny Pettersson for skilful technical assistance, and Miss Katharina Arvidsson for typing the manuscript. This study was supported by grants from the Swedish Cancer Society, the Karolinska Institute Foundations and the Swedish Medical Society.

### REFERENCES

AISENBERG, A. C. (1962) Studies on delayed hypersensitivity in Hodgkin's disease. *J. Clin. Invest.*, **41**, 1964.

AISENBERG, A. C. (1965) Quantitative estimation of the reactivity of normal and Hodgkin's disease lymphocytes with thymidine 2-C-14. *Nature*, **205**, 1233.

ASKERGREN, J. & BJÖRKHOLM, M. (1980) Postsplenectomy septicemia in Hodgkin's disease and other disorders. *Acta Chir. Scand.*, **146**, 569.

ASKERGREN, J., BJÖRKHOLM, M., HOLM, G. & 4 others (1980) Prognostic effect of early diagnostic splenectomy in Hodgkin's disease: A randomized trial. *Br. J. Cancer*, **42**, 284.

BJÖRKHOLM, M. (1978) Immunodeficiency in Hodgkin's disease and its relation to prognosis. *Scand. J. Haematol.*, Suppl. 33, 3.

BJÖRKHOLM, M., ASKERGREN, J., WEDELIN, C., HOLM, G. & MELLSTEDT, H. (1980) Blood lymphocyte functions in relation to splenic weight and tumour involvement in untreated Hodgkin's disease. *Scand. J. Haematol.*, **25**, 51.

BJÖRKHOLM, M., HOLM, G., JOHANSSON, B. & 4 others (1979) Prognostic studies in Hodgkin's disease (HD) with special reference to the influence of age. *International Society of Haematology, European and African Division, 5th Meeting*, Abstracts IV. p. 53.

BJÖRKHOLM, M., HOLM, G., MELLSTEDT, H. & JOHANSSON, B. (1975) Immunodeficiency and prognosis in Hodgkin's disease. *Acta Med. Scand.*, **198**, 275.

BJÖRKHOLM, M., HOLM, G., MELLSTEDT, H., JOHANSSON, B., ASKERGREN, J. & SÖDERBERG, G. (1977*a*) Prognostic factors in Hodgkin's disease. I. Analysis of histopathology, stage distribution and results of therapy. *Scand. J. Haematol.*, **19**, 487.

BJÖRKHOLM, M , HOLM, G. & MELLSTEDT, H. (1977*b*) Persisting lymphocyte deficiencies during remission in Hodgkin's disease. *Clin. Exp. Immunol.*, **28**, 389.

BJÖRKHOLM, M., HOLM, G. & MELLSTEDT, H. (1977*c*) Immunologic profile of patients with cured Hodgkin's disease. *Scand. J. Haematol.*, **18**, 361.

BJÖRKHOLM, M., HOLM, G., DE FAIRE, U. & MELLSTEDT, H. (1977*d*) Immunological defects in healthy twin siblings to patients with Hodgkin's disease. *Scand. J. Haematol.*, **19**, 396.

BJÖRKHOLM, M., HOLM, G., MELLSTEDT, H. & 4 others (1978*a*) Prognostic factors in Hodgkin's disease. II. The role of the lymphocyte defect. *Scand. J. Haematol.*, **20**, 306.

BJÖRKHOLM, M., HOLM, G. & MELLSTEDT, H. (1978*b*) Immunological family studies in Hodgkin's disease: Is the immunodeficiency horizontally transmitted? *Scand. J. Haematol.*, **20**, 297.

BJÖRKHOLM, M., WEDELIN, C., HOLM, G., JOHANSSON, B. & MELLSTEDT, H. (1981) Longitudinal studies of blood lymphocyte capacity in Hodgkin's disease. *Cancer*, **98**, 2010.

BONADONNA, G., DELENA, M., BANFI, A. & LATTUADA, A. (1973) Secondary neoplasms in malignant lymphomas after intensive therapy. *N. Engl. J. Med.*, **88**, 1242.

BRESLOW, N. & CROWLEY, J. (1974) A large sample study of the life table and product limit estimates under random censorship. *Ann. Statist.*, **2**, 437.

BRODY, R. S. & SCHOTTENFELD, D. (1980) Multiple primary cancers in Hodgkin's disease. *Semin. Oncol.*, **7**, 187.

BROWN, R. S., HAYNES, H. A., FOLEY, H. T., GODWIN, H. A., BERARD, C. W. & CARBONE, P. P. (1967) Hodgkin's disease. Immunologic, clinical and histologic features of 50 untreated patients. *Ann. Intern. Med.*, **67**, 291.

CANELLOS, G. P., DE VITA, V. T., ARSENEAU, J. C., WHANG-PENG, J. & JOHNSSON, R. E. C. (1975) Second malignancies complicating Hodgkin's disease in remission. *Lancet*, i, 947.

CARBONE, P. P., KAPLAN, H. S., MUSSHOFF, K., SMITHERS, D. W. & TUBIANA, M. (1971) Report of the committee on Hodgkin's disease staging classification. *Cancer Res.*, **31**, 1860.

CASE, D. C., HANSEN, J. A., CORRALES, E. & 4 others (1977) Depressed *in vitro* lymphocyte responses to PHA in patients with Hodgkin's disease in continuous long remission. *Blood*, **49**, 771.

CHASE, M. W. (1966) Delayed-type hypersensitivity and the immunology of Hodgkin's disease, with a parallel examination of sarcoidosis. *Cancer Res.*, **26**, 1097.

COX, D. R. (1972) Regression models and life tables. *J. R. Stat. Soc.*, Vol. B, **34**, 187.

DE SOUSA, M., SMITHYMAN, A. & TAN, C. (1978) Suggested models of ecotaxopathy in lymphoreticular malignancy. *Am. J. Pathol.*, **90**, 497.

ELTRINGHAM, J. R. & KAPLAN, H. S. (1973) Impaired delayed-hypersensitivity responses in 154 patients with untreated Hodgkin's disease. *Natl Cancer Inst. Monogr.*, **36**, 107

GIRARD, J. P., PAYCHÉRE, M., CUEVAS, M. & FERNANDES, B. (1977) Cell-mediated immunity in an ageing population. *Clin. Exp. Immunol.*, **27**, 85.

GOBBI, M., FIACCHINI, M., TOMASINI, I., RUGGERO, D., LAURIA, F. & TURA, S. (1977) Immunological study of patients with Hodgkin's disease in long lasting not maintained complete remission. *Boll. 1st Seiroter, Milan.* **56**, 144.

GOFFINET, D. R., GLASTEIN, E. J. & MERIGAN, T. C. (1972) Herpes Zoster-Varicella infections and lymphomas. *Ann. Intern. Med.*, **76**, 235.

GOODWIN, J. S., MESSNER, R. P., BANKHURST, A. D., PEAKE, G. T., SAIKI, J. H. & WILLIAMS, R. C., JR (1977) Prostaglandin-producing suppressor cells in Hodgkin's disease. *N. Engl. J. Med.*, **297**, 963.

GREEN, I. & CORSO, P. (1958) Experiences with skin homografting in patients with lymphoma. *Transplant. Bull.*, **5**, 427.

HERSH, E. M. & OPPENHEIM, J. J. (1965) Impaired *in vitro* lymphocyte transformation in Hodgkin's disease. *N. Engl. J. Med.*, **273**, 1006.

HOLM, G., ANGELIN, B., BJÖRKHOLM, M., ERICSSON, P., MELLSTEDT, H. & PETTERSSON, D. (1979*a*) Immunosuppressive serum factors and lymphocyte deficiency in Hodgkin's disease. *J. Clin. Immunol.*, **1**, 269.

HOLM, G., BJÖRKHOLM, M., JOHANSSON, B., MELLSTEDT, H. & LINDEMALM, C. (1981) Monocyte function in Hodgkin's disease. *Clin. Exp. Immunol.*, (in press).

HOLM, G., BJÖRKHOLM, M. & MELLSTEDT, H. (1979*b*)

Lymphocyte abnormalities and serum factors in Hodgkin's disease. In *Naturally-occurring Biological Immunosuppressive Factors and Their Relationship to Disease* Ed. Neubauer. Florida: CRC. Press, p. 3.

Holm, G., Mellstedt, H., Björkholm, M. & 4 others (1976) Lymphocyte abnormalities in untreated patients with Hodgkin's disease. *Cancer*, **37**, 751.

Holm, G., Perlmann, P. & Johansson, B. (1967) Impaired phytohaemagglutinin induced cytotoxicity *in vitro* of lymphocytes from patients with Hodgkin's disease or chronic lymphatic leukemia. *Clin. Exp. Immunol.*, **2**, 351.

Jondal, M., Holm, G. & Wigzell, H. (1972) Surface markers on human T and B lymphocytes. I. A large population of lymphocytes forming nonimmune rosettes with sheep red blood cells. *J. Exp. Med.*, **136**, 207.

Kaplan, H. S. (1980*a*) Hodgkin's disease: Unfolding concepts concerning its nature, management and prognosis. *Cancer*, **45**, 2439.

Kaplan, H. S. (1980*b*) *Hodgkin's Disease*. Cambridge Mass: Harvard Univ. Press, p. 270.

Kaplan, E. S. & Meier, P. (1958) Non-parametric estimation from incomplete observation. *Am. Stat. Assoc. J.*, **53**, 457.

Kelly, W. D., Good, R. A., Varco, R. L. & Levitt, M. (1958) The altered response to skin homografts and to delayed allergens in Hodgkin's disease. *Surg. Forum.*, **9**, 785.

King, G. W., Yanes, B., Hurtubise, P. E., Balcerzak, S. P. & LoBuglio, A. F. (1976) Immune function of successfully treated lymphoma patients. *J. Clin. Invest.*, **57**, 1451.

Levy, R. & Kaplan, H. S. (1974) Impaired lymphocyte function in untreated Hodgkin's disease. *N. Engl. J. Med.*, **290**, 181.

Lukes, R. J., Craver, L. F., Hall, T. C., Rappaport, H. & Ruben, P. (1966) Report of the nomenclature committee. *Cancer Res.*, **26**, 1311.

Miller, D. G., Lizardo, J. G. & Snyderman, R. K. (1961) Homologous and heterologous skin transplantation in patients with lymphomatous disease. *J. Natl Cancer Inst.*, **26**, 569.

Sokal, J. E. & Primikirios, N. (1961) The delayed skin test response in Hodgkin's disease and lymphosarcoma: Effect of disease activity. *Cancer*, **14**, 597.

Twomey, J. J., Laughter, A. H., Lazar, S. & Douglass, C. C. (1975) Hodgkin's disease. An immunodepleting and immunosuppressive disorder. *J. Clin. Invest.*, **56**, 467.

Twomey, J. J., Laughter, A. H., Lazar, S. & Douglass, C. C. (1976) Reactivity of lymphocytes from primary neoplasms of lymphoid tissues. *Cancer*, **38**, 740.

Twomey, J. J. & Rice, L. (1980) Impact of Hodgkin's disease upon the immune system. *Semin. Oncol.*, **7**, 114.

Wedelin, C., Björkholm, M., Holm, G., Ogenstad, S. & Mellstedt, H. (1982) Blood T-lymphocyte functions in healthy adults in relation to age. *Scand. J. Haematol.* (in press).

Weitzman, S. & Aisenberg, A. C. (1977) Fulminant sepsis after the successful treatment of Hodgkin's disease. *Am. J. Med.*, **62**, 47.

Willson, J. K. Jr, Zaremba, J. L. & Pretlow, T. G., II (1977) Functional characterization of cells separated from suspensions of Hodgkin's disease tumor cells in an isokinetic gradient. *Blood*, **50**, 783.

Young, R. C., Corder, M. P., Haynes, H. A. & De Vita, V. T. (1972) Delayed hypersensitivity in Hodgkin's disease, A study of 103 untreated patients. *Am. J. Med.*, **52**, 63.